# What Is eHealth (3): A Systematic Review of Published Definitions

**DOI:** 10.2196/jmir.7.1.e1

**Published:** 2005-02-24

**Authors:** Hans Oh, Carlos Rizo, Murray Enkin, Alejandro Jadad

**Affiliations:** ^2^Department of Health Policy Management and EvaluationUniversity of TorontoToronto ONCanada; ^1^Centre for Global eHealth InnovationUniversity Health NetworkToronto ONCanada

**Keywords:** eHealth, Internet, medical informatics, systematic review, information services, telemedicine

## Abstract

**Context:**

The term eHealth is widely used by many individuals, academic institutions, professional bodies, and funding organizations. It has become an accepted neologism despite the lack of an agreed-upon clear or precise definition. We believe that communication among the many individuals and organizations that use the term could be improved by comprehensive data about the range of meanings encompassed by the term.

**Objective:**

To report the results of a systematic review of published, suggested, or proposed definitions of eHealth.

**Data Sources:**

Using the search query string “eHealth” OR “e-Health” OR “electronic health”, we searched the following databases: Medline and Premedline (1966-June 2004), EMBASE (1980-May 2004), International Pharmaceutical Abstracts (1970-May 2004), Web of Science (all years), Information Sciences Abstracts (1966-May 2004), Library Information Sciences Abstracts (1969-May 2004), and Wilson Business Abstracts (1982-March 2004). In addition, we searched dictionaries and an Internet search engine.

**Study Selection:**

We included any source published in either print format or on the Internet, available in English, and containing text that defines or attempts to define eHealth in explicit terms. Two of us independently reviewed titles and abstracts of citations identified in the bibliographic databases and Internet search, reaching consensus on relevance by discussion.

**Data Extraction:**

We retrieved relevant reports, articles, references, letters, and websites containing definitions of eHealth. Two of us qualitatively analyzed the definitions and coded them for content, emerging themes, patterns, and novel ideas.

**Data Synthesis:**

The 51 unique definitions that we retrieved showed a wide range of themes, but no clear consensus about the meaning of the term eHealth. We identified 2 universal themes (health and technology) and 6 less general (commerce, activities, stakeholders, outcomes, place, and perspectives).

**Conclusions:**

The widespread use of the term eHealth suggests that it is an important concept, and that there is a tacit understanding of its meaning. This compendium of proposed definitions may improve communication among the many individuals and organizations that use the term.

## Introduction

During the 1990s, as the Internet exploded into public consciousness, a number of e-terms began to appear and proliferate. The terms were useful: email brought new possibilities for people to communicate rapidly and share experiences; e-commerce proposed new ways to conduct business and financial transactions through the Internet. The introduction of *eHealth* represented the promise of information and communication technologies to improve health and the health care system [1]. It too has become an indispensable term.

As with most neologisms, the precise meaning of eHealth varied with the context in which the term was used. Nevertheless, it has been fairly well understood, and is now widely used by many academic institutions, professional bodies, and funding organizations. We recognized the impossibility of finding a universally acceptable, universally applicable formal definition, yet felt that a clearer understanding of the term could be achieved by reviewing the range of proposed meanings. What is this thing called eHealth? Two previous articles in this journal have dealt with the question of how eHealth can be or should be defined [2,3]. The aim of this paper is to systematically search the literature for definitions, which have been published to date, in an attempt to answer this unanswerable question and to determine the contexts or settings in which the term has been used.

To the best of our knowledge, no such search has previously been carried out or published. We believe that a better understanding of the meaning and perspectives of eHealth could improve communication among the many individuals and organizations that use the term. For this reason, we collected, examined, and qualitatively analyzed the published proposed definitions of the term eHealth.

## Methods

### Systematic Review

We first conducted a systematic review of the peer-reviewed literature to capture as many definitions of eHealth as possible. Our inclusion criteria required that a source be published in either print format or on the Internet, be available in English, and contain text that defines or attempts to define eHealth in explicit terms.

We searched the following electronic databases: Medline and Premedline (1966-June 2004), EMBASE (1980-May 2004), International Pharmaceutical Abstracts (1970-May 2004), Web of Science (all years), Information Sciences Abstracts (1966-May 2004), Library Information Sciences Abstracts (1969-May 2004), Wilson Business Abstracts (1982-March 2004).

For each database, we used the search query string “*eHealth*”*OR “e-Health” OR “electronic health”*. In addition, we then searched dictionaries [4,5] and the Google web search engine (June 2004) which ranks retrieval by importance and relevance [6]. Because the search of Google resulted in an overwhelming number of hits, we reviewed only the first 400 results. We also refined our search by including the additional term *definition* and again reviewed the first 400 hits. We then conducted a further search using the search query string *“what is eHealth” OR “what is e-Health”*, reviewing all 358 results. We conducted our searches between February 1, 2004, and June 30, 2004. A summary of our search strategy and results is presented in Tables 1 and 2.

Two of us (HO, CR) independently reviewed titles and abstracts of citations identified in the bibliographic databases. By viewing summaries and websites of the Internet search, we reached consensus on relevance by discussion. We retrieved the relevant reports, articles, references, letters, and websites. We also manually searched the reference lists of the articles reviewed for additional relevant sources. From the hard or electronic copy of each report, we obtained the following data: author name, publication year, source, and definition (listed in Table 3). We identified and excluded duplicate definitions.

### Qualitative Analysis

Upon collection, we analyzed all the definitions and coded for content, emerging themes, patterns, and novel ideas. We used the constant comparative method described by Strauss and Corbin [7] involving open coding, axial coding, and selective coding. The constant comparative method is an iterative process of analyzing qualitative data (ie, text). Units of text (ie, words, phrases, sentences, or paragraphs) are labeled, compared, and grouped until no new categories emerge. Two of us (HO, CR) independently coded the definitions and compared results for consistency and reliability using a commercially available qualitative analytical software package (QSR NVivo v2.0).

## Results

### Systematic Review

In total, we scanned 1209 abstracts and reviewed 430 citations from the bibliographic databases. From these we collected 10 different definitions for the term eHealth (Table 1). From the Google search, we reviewed 1158 sites and identified 41 additional unique definitions (Table 2).

The definitions that we found were as short as 3 words [8] or as long as 74 words [9] (Table 3). We identified 2 universal themes (health and technology) and 6 less generally mentioned themes (commerce, activities, stakeholders, outcomes, place and perspectives) (Table 4).

**Table 1:** Summary of database searches

**Database (time)**	**Citations**	**Articles Reviewed**	**Unique Definitions**
MEDLINE (1966-June 2004)	493	157	10
EMBASE (1975-2003)	218	73	0
International Pharmaceutical Abstracts	16	3	0
Information Sciences & Library Sciences Abstracts	61	15	0
Web of Science	217	77	0
Wilson Business Abstracts	204	105	0
**Total**	**1209**	**430**	**10**

**Table 2:** Summary of Google searches

**Search Query**	**Citations**	**Sources Reviewed**	**Unique Definitions**
“eHealth” OR “e-Health” OR “electronic health”	960000	400	0
“eHealth” OR “e-Health” AND definition	77000	400	9
“what is eHealth” OR “what is e-Health”	358	358	32
**Total**	**1037358**	**1158**	**41**

**Table 3:** Definitions (verbatim quotations) of eHealth presented in chronological order

	**Year**	**Source** (M = Medline, W = Wilson Business Abstracts, G = Google)	**Definition**
1	1999	Mitchell [42] (G)	A new term needed to describe the combined use of electronic communication and information technology in the health sector. The use in the health sector of digital data – transmitted, stored and retrieved electronically – for clinical, educational and administrative purposes, both at the local site and at a distance.
2	1999	Loman - First Consulting Group [12] (G)	E-health – the application of e-commerce to healthcare and pharmaceuticals
3	2000	JHITA [13] (G)	Internet-related healthcare activities
4	2000	McLendon [14] (M)	Ehealth refers to all forms of electronic healthcare delivered over the Internet, ranging from informational, educational and commercial "products" to direct services offered by professionals, non-professionals, businesses or consumers themselves. Ehealth includes a wide variety of the clinical activities that have traditionally characterized telehealth, but delivered through the Internet. Simply stated, Ehealth is making healthcare more efficient, while allowing patients and professionals to do the previously impossible.
5	2000	Medical Business News [46] (G)	E-Health is a convergence between the Internet and the health care industry to provide consumers with a wide variety of information relating to the health care field
6	2000	GJW Government Relations [52](G)	A wide-ranging area of social policy that uses new media technologies to deliver both new and existing health outcomes
7	2000	Oracle Corporation [15] (G)	Healthcare transactions, encounters, messaging, or care provision occurring electronically.
8	2000	DeLuca, Enmark - Frontiers of Medicine [16] (W) (M)	E-health is the embryonic convergence of wide-reaching technologies like the Internet, computer telephony/interactive voice response, wireless communications, and direct access to healthcare providers, care management, education, and wellness.
9	2000	Pretlow [17] (G)	E-health is the process of providing health care via electronic means, in particular over the Internet. It can include teaching, monitoring ( e.g. physiologic data), and interaction with health care providers, as well as interaction with other patients afflicted with the same conditions.
10	2001	Baur, Deering and Hsu [11] (G)	The most broad term is ehealth, with refers to the use of electronic technologies in health, health care and public health. (...) The various functions of ehealth [are]: (...) reference (electronic publishing, catalogues, databases); self-help/self-care (online health information, support groups, health risk assessment, personal health records), Plan/provider convenience services (online scheduling, test and lab results, benefit summaries), Consultation and referral (doctor-patient or doctor-doctor consultation via telemedicine systems, remote readings of digital image and pathology samples), E-health commerce (sales of health related product and services) [and] Public health services (automated data collection, data warehouses, online access to population survey data and registries, advance detection and warning systems for public health threats). (...) This chapter uses the term ehealth to refer to the broadest possible range of interactive technologies applied to health and health care.
11	2001	Orlikoff & Totten [18] (M)	The use of the Internet and related information systems and technology in all aspects of health care.
12	2001	Eysenbach [3] (M)	e-health is an emerging field in the intersection of medical informatics, public health and business, referring to health services and information delivered or enhanced through the Internet and related technologies. In a broader sense, the term characterizes not only a technical development, but also a state-of-mind, a way of thinking, an attitude, and a commitment for networked, global thinking, to improve health care locally, regionally, and worldwide by using information and communication technology
13	2001	Blake [43] (M)	The combined use of electronic communication and information technology in the health sector. It is important to note that e-health is much more than business transactions. It encompasses everything from digital data transmission to purchase orders, lab reports, patient histories and insurance claims.
14	2001	Strategic Health Innovations [19] (G)	The use of information technology in the delivery of health care.
15	2001	Robert J Wood Foundation [20] (G)	EHealth is the use of emerging information and communication technology, especially the Internet, to improve or enable health and health care.
16	2001	Wysocki [21] (G)	e-Health refers to all forms of electronic healthcare delivered over the Internet, ranging from informational, educational and commercial "products" to direct services offered by professionals, non-professionals, businesses or consumers themselves
17	2001	JP Morgan Partners [45] (G)	The health care industry's component of business over the Internet
18	2001	Ontario Hospital eHealth Council [22] (G)	EHealth is a consumer-centred model of health care where stakeholders collaborate utilizing ICTs including Internet technologies to manage health, arrange, deliver, and account for care, and manage the health care system.
19	2001	Tieman [55] (M)	E-health is all that's digital or electronic in the healthcare industry
20	2001	DeLuca, Enmark [61] (M)	E-health is the electronic exchange of health-related data across organizations, although every health care constituent approaches e-health differently.
21	2001	Ball – HIMSS [47] (G)	Internet technologies applied to the healthcare industry
22	2002	Health e-Technologies Initiative [23] (G)	The use of emerging interactive technologies (i.e., Internet, interactive TV, interactive voice response systems, kiosks, personal digital assistants, CD-ROMs, DVD-ROMs) to enable health improvement and health care services.
23	2002	Grantmakers in Health [24] (G)	Use of ICT, especially (but not only) the Internet to enable health and health care.
24	2002	Kirshbaum [25] (G)	There are many different definitions of eHealth Electronic connectivity vehicle for improving the efficiency and effectiveness of healthcare delivery Enabling consumers/patients to be better informed about their healthcare Enabling providers to deliver better care in more efficient ways
25	2002	Wyatt and Liu [51] (M)	The use of internet technology by the public, health workers, and others to access health and lifestyle information, services and support; it encompasses *telemedicine, telecare*, etc.
26	2003	Staudenmeir - Arthur Anderson [26] (G)	Any use of the Internet or related technology to improve: the health and wellness of the population; the quality of healthcare services and outcomes; efficiencies in healthcare services or administration
27	2003	COACH [39] (G)	The leveraging of the information and communication technology (ICT) to connect provider and patients and governments; to educate and inform health care professionals, managers and consumers; to stimulate innovation in care delivery and health system management; and, to improve our health care system.
28	2003	Rx2000 [9] (G)	eHealth signifies a concerted effort undertaken by some leaders in healthcare and hi-tech industries to harness the benefits available through convergence of the Internet and healthcare. Access, cost, quality and portability have been concerns in the health care arena. It's evident from many recent surveys that both health consumers and healthcare professionals are frustrated with the maze of health care delivery. Some, therefore, are turning to the Internet for answers and cost effective solutions.
29	2003	Beaulieu & Beinlich - First Consulting Group [27] (G)	eHealth (ē´helth), n. 1. The application of Internet principles, techniques and technologies to improve healthcare. 2. New way of conducting the business of healthcare enabling stronger and more effective connections among patients, doctors, hospitals, employers, brokers, payers, laboratories, pharmacies, and suppliers. 3. The “customer facing” e-revolution in healthcare. [1999]
30	2003	eEurope - eHealth2003 [53] (G)	The application of information and communication technologies (ICT) across the whole range of functions which one way or another, affect the health of citizens and patients.
31	2003	Decker – HealthVision [28] (G)	Corporate strategy and using the power of the Internet and emerging technology to redefine the delivery of health care.
32	2003	Miller - athealth.com [29] (G)	E-health means any form of healthcare information made available over the Internet.
33	2003	Telehealth Victoria [30] (G)	Term that is used to describe most aspects of healthcare delivery or management that is enabled by information technology or communications
34	2003	Ebrunel.com [31] (G)	The provision of healthcare services available through the Internet - and particularly to the rash of health related web sites.
35	2003	Regional Office for the Eastern Mediterranean - World Health Organization [44] (G)	E-health is a new term used to describe the combined use of electronic communication and information technology in the health sector OR is the use, in the health sector, of digital data-transmitted, stored and retrieved electronically-for clinical, educational and administrative purposes, both at the local site and at a distance
36	2003	www.avienda.co.uk [32] (G)	A generic field of information and communications technologies used in medicine and healthcare.
37	2003	Brommey [33]	The use of electronic information and communications technologies to provide and support health care wherever the participants are located
38	2003	Southwest Medical Group [34] (G)	e-health is an emerging field focused on medical information and health care services delivered or enhanced through advanced Internet or related technologies. In a broader sense, the term extends the scope of health care beyond its conventional boundaries. Conceptually, e-health enables patients to easily obtain medical related services online from health care providers
39	2003	HMS Europe [40] (G)	The practice of leveraging the Internet to connect caregivers, healthcare systems and hospitals with consumers
40	2003	Nova Scotia Telehealth Network [62] (G)	E-health is a broad term to describe the accessing of information, products and services on "e-health" sites
41	2003	Strengthening Support for Women with Breast Cancer [35] (G)	The use of information and communication technology (ICT) to enhance health care.
42	2003	Vigneault [10] (G)	The development and evolution of technical tools to support program delivery
43	2003	Policy on ICT Security [50] (G)	Using the Internet and other electronic channels to access and delivery health and lifestyle information and services
44	2003	Health systems group [49] (G)	eHealth is health promotion delivered and managed over the Internet
45	2003	Marcus and Fabius [8] (G)	Ehealth is connectivity
46	2003	Silber [54] (G)	eHealth is the application of information and communications technologies (ICT) across the whole range of functions that affect health.
47	2003	Ehealth Technologies [36] (G)	The use of emerging information and communication technology, especially the Internet, to improve or enable health and healthcare thereby enabling stronger and more effective connections among patients, doctors, hospitals, payors, laboratories, pharmacies, and suppliers
48	2003	International Telecommunication Union [41] (G)	Encompasses all of the information and communication technologies (ICT) necessary to make the health system work
49	2003	Baker [48] Modified from Gott (1993) (G)	The promotion and facilitation of health and well-being with individuals and families and the enhancement of professional practice by the use of information and communication technology
50	2004	Sternberg [37] (M)	New business models using technology to assist healthcare providers in caring for patients and providing services.
51	2004	Watson [38] (M)	The integration of the internet into health care.

**Table 4:** Themes found in definitions of eHealth

	**Year**	**Source**(M = Medline, W = Wilson Business Abstracts, G = Google)	**Health**	**Technology**	**Stakeholders**	**Activities**	**Attitudes**	**Place**	**Outcomes**	**Commerce**
1	1999	Mitchell [42] (G)	X	X		X		X		
2	1999	Loman - First Consulting Group [12] (G)	X							X
3	2000	JHITA [13] (G)	X	X		X				
4	2000	McLendon [14] (M)	X	X	X	X	X		X	
5	2000	Medical Business News [46] (G)	X	X	X		X			X
6	2000	GJW Government Relations [52](G)	X	X		X	X		X	
7	2000	Oracle Corporation [15] (G)	X			X				
8	2000	DeLuca, Enmark - Frontiers of Medicine [16] (W) (M)	X	X	X					
9	2000	Pretlow [17] (G)	X	X	X	X				
10	2001	Baur, Deering and Hsu [11] (G)	X	X						
11	2001	Orlikoff & Totten [18] (M)	X	X						
12	2001	Eysenbach [3] (M)	X	X	X	X	X	X	X	X
13	2001	Blake [43] (M)	X	X		X				X
14	2001	Strategic Health Innovations [19] (G)	X	X		X				
15	2001	Robert J Wood Foundation [20] (G)	X	X					X	
16	2001	Wysocki [21] (G)	X	X	X	X				X
17	2001	JP Morgan Partners [45] (G)	X	X						X
18	2001	Ontario Hospital eHealth Council [22] (G)	X	X	X	X	X			
19	2001	Tieman [55] (M)	X	X						X
20	2001	DeLuca, Enmark [61] (M)	X	X	X					
21	2001	Ball – HIMSS [47] (G)	X	X						X
22	2002	Health e-Technologies Initiative [23] (G)	X	X		X			X	
23	2002	Grantmakers in Health [24] (G)	X	X		X				
24	2002	Kirshbaum [25] (G)	X		X	X			X	
25	2002	Wyatt and Liu [51] (M)	X	X	X	X				
26	2003	Staudenmeir - Arthur Anderson [26] (G)	X	X					X	
27	2003	COACH [39] (G)	X	X	X	X			X	
28	2003	Rx2000 [9] (G)	X	X	X	X			X	
29	2003	Beaulieu & Beinlich - First Consulting Group [27] (G)	X	X	X	X	X		X	X
30	2003	eEurope - eHealth2003 [53] (G)	X	X	X				X	
31	2003	Decker – HealthVision [28] (G)	X	X			X			X
32	2003	Miller - athealth.com [29] (G)	X	X						
33	2003	Telehealth Victoria [30] (G)	X	X		X				
34	2003	Ebrunel.com [31] (G)	X	X		X				
35	2003	Regional Office for the Eastern Mediterranean - World Health Organization [44] (G)	X	X		X		X		
36	2003	www.avienda.co.uk [32] (G)	X	X						
37	2003	Brommey [33]	X	X	X	X				
38	2003	Southwest Medical Group [34] (G)	X	X	X	X		X		
39	2003	HMS Europe [40] (G)	X	X	X					
40	2003	Nova Scotia Telehealth Network [62] (G)	X			X				
41	2003	Strengthening Support for Women with Breast Cancer [35] (G)	X	X					X	
42	2003	Vigneault [10] (G)		X		X				
43	2003	Policy on ICT Security [50] (G)	X	X		X				
44	2003	Health systems group [49] (G)	X	X		X				
45	2003	Marcus and Fabius [8] (G)					X			
46	2003	Silber [54] (G)	X	X						
47	2003	Ehealth Technologies [36] (G)	X	X	X	X			X	
48	2003	International Telecommunication Union [41] (G)	X	X						
49	2003	Baker [48] Modified from Gott (1993) (G)	X	X	X	X			X	
50	2004	Sternberg [37] (M)	X	X	X	X				X
51	2004	Watson [38] (M)	X	X						

### Qualitative Analysis

Not surprisingly, all the definitions included the theme of health. The word *health* per se was used in almost all 51 definitions collected (only two did not include it) [8,10]. Most commonly, the word *health* was used in relation to health services delivery (eg, health care [3,11-38], health system [39-41], health sector [16,22,42-44] or health industry [9,45-47]) which suggests that eHealth may refer more to services and systems rather than to the health of people. Wellness as a concept was used only 5 times (namely, wellness [3], public health [26], health and wellness [48], health and well-being [49], and health promotion [13]).

All the definitions also referred to technology, either explicitly or implicitly. The word *Internet* was explicitly mentioned in 27 of the 51 definitions [3, 9, 11, 13, 14, 16-18, 20-24, 26-29, 31, 34, 38, 40, 45-47, 49-51]; 4 of them used *Internet* as an adjective (Internet-related [13], Internet technologies [27, 51], or Internet principles [27]) rather than as a noun. Some authors listed specific technologies such as interactive television [23], personal digital assistants [23], CD-ROMs/DVD [23] or Internet telephony [16]. Others referred to technology in more general terms (eg, new media [52], information and communication technologies [19, 20, 22, 24, 30, 32, 33, 35, 36, 39, 41-44, 48, 53, 54], and Internet-related technologies [3,11,18,26,27,34]). Only 1 definition [38] used the term *integration*.

In 11 definitions, [3,12,21,27,28,37,43,45-47,55] eHealth was referred to in terms of commerce, suggesting that eHealth is “health care's component of business over the Internet” [45], the “application of e-commerce to health care and pharmaceuticals” [12], or as “new business models using technology” [37]. Others associated eHealth with activities such as managing [22], educating [39], arranging [22], connecting [39], obtaining [34], providing [33], redefining [28], supporting [33], using [42], assisting [37] and accessing [51]. The stakeholders most often mentioned were health care providers (doctors [27,36], health care providers [16,37], health care professionals [34,39], health workers [51], managers [39], and caregivers [40]). The public is mentioned as public [51], patients [17,25,27,34,39,53], consumers [14,21,25,39], non-professionals [14,21,46], and citizens [53]. Governments [39], employers [27], and payers [27] are also listed as potentially benefiting from eHealth.

While most of the definitions concentrated on the process of care, about one quarter of them focused on the outcomes to be expected. These definitions mentioned improving and increasing the cost-effectiveness of health care [9] and making processes more efficient [14,25,26]. Others suggested that eHealth could solve problems related to access to care, cost, quality, and portability of health care services [9].

While the actual word *place* was not used in any of the definitions, some authors referred to the concepts of distance, geography, and location. One definition describes the impact of eHealth as local, regional, and worldwide [3]. Another describes eHealth as taking place both at the local site and at a distance [42]. A third suggests that distance and place no longer remain barriers, as eHealth is “to provide and support health care wherever the participants are located” [33].

Finally, other definitions suggest that eHealth represents a new perspective on health care. One author describes eHealth as a “state-of-mind, a way of thinking, an attitude, and a commitment for networked, global thinking” [3]. Another source describes eHealth as a “consumer-centered model of health where stakeholders collaborate” [22].

## Discussion

The term eHealth encompasses a set of disparate concepts, including health, technology, and commerce. The 51 unique published definitions that we found included these concepts with varying degrees of emphasis. All specifically mentioned health and the technology involved. Many noted the varying stakeholders, the attitudes encompassed, the role of place and distance, and the real or potential benefits to be expected from eHealth.

Health, as used in these definitions, usually referred explicitly to health care as a process, rather than to health as an outcome. This is as expected; there is no consensus on the meaning of the word *health* per se, the definitions of which range from a narrowly construed “converse of disease or infirmity or when disease or infirmity is absent” [56] to the all-encompassing World Health Organization's “health is a state of complete physical, mental, and social well being and not just the absence of disease or infirmity” [57].

In the definitions of eHealth we found, technology was viewed both as a tool to enable a process/function/service and as the embodiment of eHealth itself (eg, a health website on the Internet). We were pleased to note that technology was portrayed as a means to expand, to assist, or to enhance human activities, rather than as a substitute for them. Surprisingly few of the published definitions referred explicitly to the commercial aspects of eHealth (Table 4).

The overwhelming understanding of eHealth reflects an attitude of optimism. All definitions had positive connotations and included terms such as benefits [9], improvement [3,20,23,26,27], enhancing [34,35,48], efficiency [3,25], and enabling [20,23,25,27,36]. One definition suggests that eHealth allows patients and professionals to "do the previously impossible" [14]. None of the published definitions suggests that eHealth may have any adverse, negative, harmful, or disadvantageous effects.

In this review, we do not report the frequency with which certain definitions were used by others, or the impact of each definition. The most commonly cited definition on the Internet is Eysenbach's [3] which was adopted or referred to by at least 87 websites on the Internet. Mitchell's definition [42] was used by a handful of others. There were many variations on the definition that characterizes eHealth as the "use of information technology in the delivery of health care" [19]. Most definitions implied that theirs was “the” definition.

In a perfectly logical language, as envisioned by Ludwig Wittgenstein in his early years [58], each word would have a specific and clear meaning. The philosopher himself recognized that such an idealized language could not be achieved in real life; he concluded his classic book, *Tractatus Logico-Philosophicus*, with "My propositions serve as elucidations in the following way: anyone who understands me eventually recognizes them as nonsensical, when he has used them—as steps—to climb up beyond them. (He must, so to speak, throw away the ladder after he has climbed up it.)" [58]. In his later work, *Philosophical Investigations* [59], Wittgenstein compares words in a language to tools in a toolbox, saying that their functions are varied according to the needs of the speaker much like the tools in a toolbox are varied according to the needs of the repairman. Their functional differences are what make them practical, and in the case of words this difference is usage. The way in which a word is used is what makes it useful in the language; a particular usage of a word gives the word its special authority in that situation [60]. For this reason we have not yielded to the temptation (nor do we have the chutzpah) to attempt another “better” definition of eHealth. The widespread use of the term suggests that eHealth is an important concept, and the term is a useful “tool” to express that concept. It is generally understood despite the lack of a precise definition. The variations among the proposed definitions reflect the various perspectives, settings and contexts in which *eHealth* is used; they round and enhance our understanding of the concept.

In this systematic review and qualitative analysis of the definitions, we have completed only a first step in research on the evolving meaning of eHealth. It is an essential first step because it tells us how the current literature defines the term. We hope, and believe, that this compilation of existing definitions can be a useful resource to facilitate communication, discussion, and stimulate further research.

Questions remain about how the differing concepts and understandings of the term eHealth affect different stakeholders. What do people expect from eHealth? Do patients want eHealth? Do health care providers want eHealth? How does eHealth change the relationships, understandings, and interactions within the health care system? Time, patience, and further research will provide at least provisional answers to these questions, and to the myriad of questions still unasked.
